# Clinical and pathohistological characteristics of Alport spectrum disorder caused by *COL4A4* mutation c.193-2A>C: a case series

**DOI:** 10.3325/cmj.2021.62.204

**Published:** 2021-06

**Authors:** Petar Šenjug, Tamara Nikuševa, Martić, Marija Šenjug Perica, Maja Oroz, Matija Horaček, Kristina Gotovac, Jerčić, Krešimir Galešić, Danica Galešić, Ljubanović

**Affiliations:** 1 Department of Nephropathology and Electron Microscopy, Department of Pathology and Cytology, Dubrava University Hospital, Zagreb, Croatia; 2 Department of Biology, Zagreb University School of Medicine, Zagreb, Croatia; 3 Children's Hospital Srebrnjak, Zagreb, Croatia; 4 Dr Fran Mihaljević University Hospital for Infectious Diseases, Zagreb University School of Medicine, Zagreb, Croatia; 5 Institute of Pathology, Zagreb University School of Medicine, Zagreb, Croatia; 6 Department of Neurology, University Hospital Center Zagreb, Zagreb, Croatia; 7 Department of Nephrology, Dubrava University Hospital, Zagreb, Croatia

## Abstract

**Aim:**

To present the pathohistological and clinical characteristics of five Croatian families with Alport spectrum disorders caused by splice acceptor pathogenic variant c.193-2A>C in *COL4A4* at the genomic position chr2:227985866.

**Methods:**

The study enrolled five probands with kidney biopsy analysis and five family members. Mutation screening was performed with Illumina MiSeq platform. The pathogenic variant was confirmed with standard dye-terminator sequencing.

**Results:**

The only homozygous patient, aged two, had proteinuria and hematuria with preserved kidney function and no extrarenal manifestations. This patient had changes characteristic for Alport syndrome observed on electron microscopy of the kidney biopsy. In the heterozygous group, six patients had hematuria, four biopsied probands had proteinuria, and only one had moderately reduced kidney function. Heterozygous probands had variable kidney biopsy findings. Three patients had thin glomerular basement membrane nephropathy visible on electron microscopy and focal segmental glomerulosclerosis on light microscopy, two of them with focal lamellation on electron microscopy. One heterozygous patient had changes characteristic for Alport syndrome on electron microscopy without focal segmental glomerulosclerosis.

**Conclusion:**

The homozygous patient had hematuria and proteinuria with preserved kidney function. The heterozygous patients presented with reasonably mild clinical phenotype and variable pathohistological findings.

Alport syndrome (AS) is a structural disorder of the glomerular basement membrane (GBM). Its genetic basis lies in the diverse mutations of *COL4A3*, *COL4A4,* and *COL4A5* genes and it phenotypically manifests as a progressive nephropathy with hematuria, ultrastructural changes of the GBM, sensorineural hearing impairment, and eye abnormalities (1-5). The most frequent mutations (85%) are *COL4A5* mutations, resulting in X-linked AS (6). Individuals with autosomal recessive AS (ARAS), caused by two mutations in *COL4A3* and/or *COL4A4*, have similar clinical features to men with X-linked AS (7,8). The type of mutation affects disease phenotype and manifestation. The phenotype is usually severe both in men and women, with early onset of end-stage renal disease (ESRD) and frequent extrarenal disorders (9,10).

The spectrum of AS disorders has recently been expanded (11). Naming and describing individuals with heterozygous *COL4A3* and *COL4A4* mutations is still a matter of debate (10,12,13). A number of studies showed a correlation between thin glomerular basement membrane nephropathy (TBMN) with the heterozygosity for *COL4A3* or *COL4A4* mutation and benign familial hematuria (2,14-25). However, a variable proportion of *COL4A3* or *COL4A4* carriers progress to proteinuria, hypertension, and ESRD, which raises the question of the nomenclature of autosomal dominant AS (ADAS) (14,25-31). Some scientists advocate the use of the term ADAS, others continue to use the term TBMN, while a Cyprus research group uses the term late-onset Alport nephropathy (10,12,13,32). The rationale behind ADAS nomenclature for heterozygous *COL4A3* or *COL4A4* patients lies in the presence of thin GBM in the kidney biopsy specimens of patients with X-linked AS and ARAS and the heterozygous carriers of *COL4A3* or *COL4A4* mutation (10). The authors suggest that this approach would improve clinical and diagnostic evaluation, with the possibility of ESRD rate reduction and treatment optimization (10). There are also rationales behind the use of the term TBMN. Savige et al (13) stated that most of heterozygous *COL4A3* and *COL4A4* carriers show either no decline in kidney function or show only mild decline with inconstant progression to ESRD and hearing impairment. The authors also argue that there is no unmistakable evidence that one mutation in *COL4A3* or *COL4A4* gene without disease modifying factors can be responsible for the characteristic ultrastructural signs of AS, hearing impairment, or eye abnormalities (13). Furthermore, in other genetic diseases autosomal dominant (AD) term is not used for the carriers of autosomal recessive (AR) disease because it can lead to the diagnosis of AD and AR disease in different members of the same family (13). However, there are emerging reports of autosomal dominant Alport spectrum disorders, especially in the cases that are hard to diagnose clinicopathologically (33). Here, we present the pathohistological and clinical characteristics of disorders caused by splice site mutation c.193-2A>C in *COL4A4* at the genomic position chr2:227985866.

## Patients and methods

### Patients

This study is a part of the research project Genotype-Phenotype Correlation in Alport’s Syndrome and Thin Glomerular Basement Membrane Nephropathy funded by the Croatian Science Foundation. Five probands and their five family members were enrolled. The inclusion criterion for proband selection was kidney biopsy with glomerular changes on electron microscopy (EM) suggestive of AS or TBMN. The patients were selected by a retrospective review of the renal biopsy registry of the Department of Nephropathology and Electron Microscopy, Dubrava University Hospital Zagreb, covering the period from 2003 to 2019. All available clinical data, including the information about patients’ and family medical history, onset of disease, kidney function (estimated glomerular filtration rate calculated by Chronic Kidney Disease Epidemiology Collaboration [CKD-EPI] equation, where values of 90 mL/min/1.73m^2^ or above were considered as preserved kidney function) and information about ocular abnormalities and sensorineural hearing loss was collected. An expert in clinical genetics conducted counselling with all probands, created family pedigrees, and identified family members at risk, who were later included in the study. All probands and family members gave a written consent for study participation. All procedures were performed in accordance with the ethical standards of the institutional research committee and the 1964 Helsinki Declaration and its later amendments or comparable ethical standards. The project Genotype-Phenotype Correlation in Alport’s Syndrome and Thin Glomerular Basement Membrane Nephropathy was approved by the Ethics Committee of the University of Zagreb School of Medicine.

### Methods

Probands and family members underwent mutation screening with Illumina MiSeq platform (Illumina, San Diego, CA, USA). Truseq Custom Amplicon Low Input kit was designed (Illumina) for re-sequencing of *COL4A3*, *COL4A4,* and *COL4A5* genes. This custom-made panel includes 378 primer pairs that amplify the coding regions and flanking splice regions and generate amplicons ranging in size from 225 bp to 275 bp. Each sample was barcoded for multiplexing. The coverage for all exons was 99.09%. The quality of the libraries was assessed with the Agilent Bioanalyzer HS DNA Kit (Agilent Technologies, Santa Clara, CA, USA), showing correct size and concentration of the samples. The amplified libraries were pooled and sequenced on MiSeq Nano Flow Cell (Illumina). FastQ files generated by sequencing were subsequently submitted for analysis. The mean coverage depth of all amplicons was 270 × . For bioinformatical analysis, Illumina VariantStudio software was used. All variants were assigned a number in available databases, including the NCBI dbSNP138 and ClinVar (34). Splice acceptor pathogenic variant c.193-2A>C found in *COL4A4* at the genomic position chr2:227985866 (variant described according to reference genome GRCh37) was confirmed with standard dye-terminator sequencing. Sanger sequencing was performed on ABI310 (Applied Biosystems) with BigDye v1.1 chemistry (Thermo Fisher Scientific, Waltham, MA, USA). The results were visualized with Vector NTI Software (Thermo Fisher Scientific, Waltham, MA, USA).

## Results

### Patients’ characteristics

The summary of patients’ clinical characteristics is shown in Table 1, while the summary of probands’ kidney biopsy findings is shown in Table 2.

**Table 1:** Probands’ and family members’ clinical characteristics

Patient	Sex	Age	Hematuria	Proteinuria (g/24h)	Kidney function (eGFR*)	Hearing loss	Ocular abnormalities	Hypertension
Family HR1								
HR 1.1.	M	2	Yes	0.60	Preserved	No	No	No
HR 1.2.	M	37	Yes	No	Preserved	NA	NA	NA
HR 1.3.	F	35	No	No	Preserved	NA	NA	NA
Family HR2								
HR 2.1.	M	58	Yes	2.34	Preserved	NA	NA	Yes
HR 2.2.	M	36	Yes	No	Preserved	NA	NA	NA
HR 2.3.	M	33	No	No	Preserved	NA	NA	NA
Family HR3								
HR 3.1.	M	26	Yes	0.30	Preserved	No	No	Yes
Family HR4								
HR 4.1.	F	60	Yes	2.3	Preserved	NA	NA	Yes
Family HR5								
HR 5.1.	F	58	Yes	1.14	Moderately reduced (45 mL/min/1,73m^2^)	NA	NA	Yes
HR 5.2.	M	19	No	No	Preserved	NA	NA	NA

*eGFR – estimated glomerular filtration rate using Chronic Kidney Disease Epidemiology Collaboration (CKD-EPI) equation.

**Table 2:** Probands’ kidney biopsy findings*

Patient	Sex	Age	Pathohistological diagnosis	Segmental glomerulosclerosis	Arteriolar hyalinosis	IFTA (%)	GBM thickness, average (min-max), SD	Lamellation of GBM	Thickening and thinning of GBM	Podocyte foot process effacement
Family HR1										
HR 1.1.	M	2	AS	No	No	1	167 (61-390), 65	Extensive	Yes	Focal
Family HR2										
HR 2.1.	M	58	TBMN + FSGS	Yes	Marked	25	186 (96-375), 58	Focal	Yes	10%
Family HR3										
HR 3.1.	M	26	AS	No	No	10	349 (70-972), 216	Extensive	Yes	No
Family HR4										
HR 4.1.	F	60	TBMN + FSGS	Yes	Mild	15	205 (144-288), 52	Focal	No	25%
Family HR5										
HR 5.1.	F	58	TBMN + FSGS	Yes	Marked	30	175 (96-363), 71	No	No	No

*Abbreviations: AS – Alport syndrome; TBMN + FSGS – thin glomerular basement membrane nephropathy combined with focal segmental glomerulosclerosis; IFTA – interstitial fibrosis and tubular atrophy; GBM – glomerular basement membrane.

**Figure 1:**
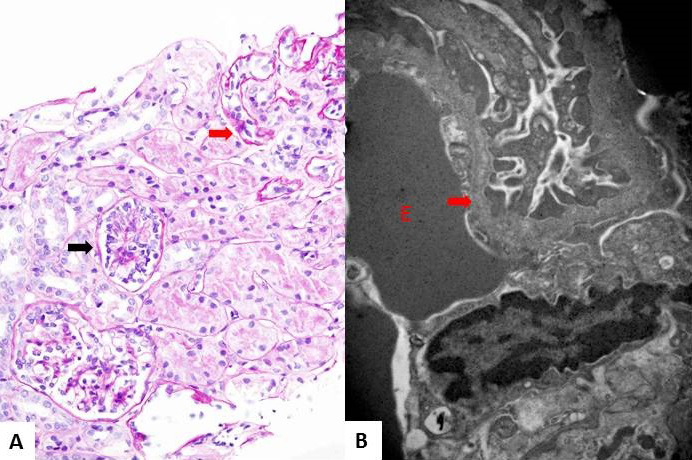
A kidney biopsy specimen of the proband HR 1.1. (**A**) The kidney cortex with an immature glomerulus (black arrow) and a small focus of interstitial fibrosis and tubular atrophy (red arrow). Periodic Acid-Schiff stain, magnification ×200. (**B**) Glomerular basement membrane with lamellation (arrow). E – erythrocyte. Transmission electron microscopy, magnification ×15,000.

**Figure 2:**
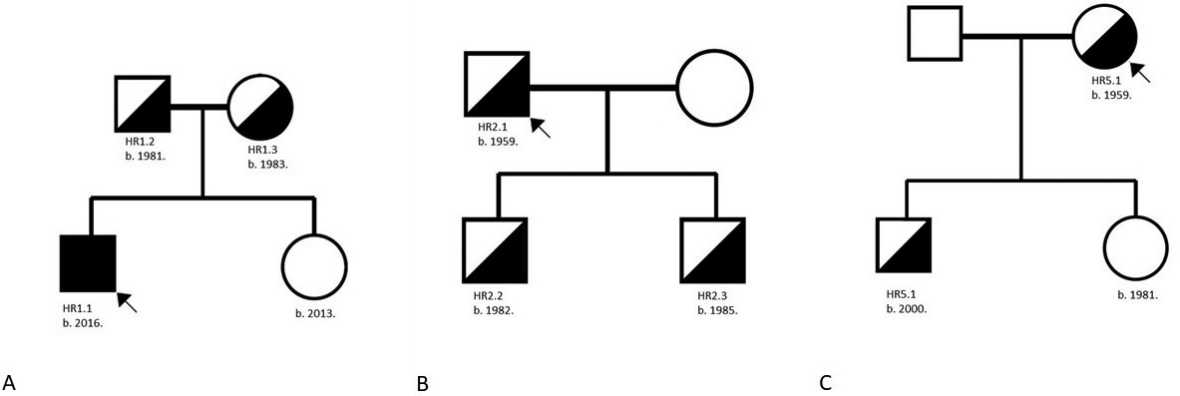
Family pedigrees of the three included families. (**A**) Family HR1. Homozygous male proband and his heterozygous parents. (**B**) Family HR2. Heterozygous male proband and his two heterozygous sons. (**C**) Family HR5. Heterozygous female proband, her heterozygous son and a healthy daughter. Arrow – proband, circle – female, square – male, black – homozygous patient, white – healthy individual, black and white – heterozygous patient.

As we previously reported (35), the first proband (HR 1.1.) was a boy aged two years and two months referred to the Nephrology Department of Children’s Hospital Zagreb due to hematuria and proteinuria. The patient experienced a delay in psycho-motoric development and megalencephaly. Extensive workup performed at 10 months revealed karyogram 46 XY and negative tests for fragile X. Organic acids in urine, homocystein, B12 and folic acid, acyl-carnitine profile, and amino acids in urine and serum were within the reference range. Brain magnetic resonance imaging was unremarkable. Megalencephaly was described as familial benign megalencephaly (his father had head circumference above the 95th centile). At the age of 1 year and 2 months, macrohematuria and proteinuria were recorded for the first time. Protein/creatinine was 284 mg/mmol; afterwards he had persistent proteinuria and microhematuria. At the age of two, he had protein/creatinine 189 mg/mmol and microhematuria. Tonal audiogram was unremarkable; eye exam did not reveal anterior lenticonus. Renal biopsy on light microscopy (LM) showed the kidney cortex with 69 glomeruli, one of which was globally sclerosed. Immature and partly immature glomeruli made 30% of all glomeruli (Figure 1A). There was one small focus of interstitial fibrosis and tubular atrophy, affecting 1% of the cortical parenchyma (Figure 1A). Changes characteristic for AS with areas of lamellation and basket-weave appearance of the GBM were present on EM (Figure 1B). After starting 6 mg/m^2^ ramipril, proteinuria decreased and protein/creatinine was 73 mg/mmol, while microhematuria persisted. Both parents were heterozygous for the mutation. The father (HR 1.2.), aged 37, presented only with microhematuria (detected only after retesting), and the mother (HR 1.3.), aged 35, showed no signs of disease (no hematuria, no proteinuria, and preserved kidney function) (35). His older sister (age 5) was negative for the mutation (Figure 2A).

In the years following our initial report on this mutation, we identified four more probands with the same mutation.

**Figure 3:**
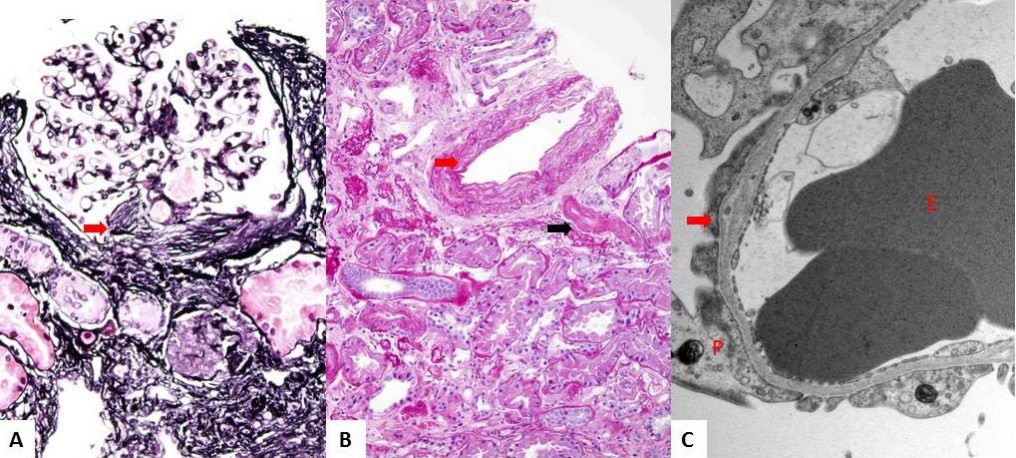
A kidney biopsy specimen of the proband HR 2.1. (**A**) A glomerulus with perihilar segmental sclerosis (arrow), Jones methamine silver stain, magnification ×400. (**B**) Marked arteriolar hyalinosis (black arrow) and mild arterial fibrointimal thickening (red arrow). Periodic Acid-Schiff stain, magnification ×200. (**C**) Glomerular capillary loop with thin glomerular basement membrane and focal lamellation (arrow). E – erythrocyte, P – podocyte. Transmission electron microscopy, magnification ×8000.

The second proband (HR 2.1.) was a 58-year-old man who presented with hematuria and proteinuria of 2.34 g in 24-hour urine, preserved kidney function (estimated glomerular filtration rate of 99 mL/min/1.73m^2^ by CKD-EPI equation), and hypertension. Perihilar focal segmental glomerulosclerosis (FSGS) in one glomerulus was observed on LM (Figure 3A); 35% of glomeruli were globally sclerotic, while others were enlarged. Interstitial fibrosis and tubular atrophy were present in 25% of cortical parenchyma. Arterioles showed marked hyalinosis, while arteries showed mild fibrointimal thickening (Figure 2B). EM revealed thin GBM (average thickness 186 nm) with discrete lamellation in the areas of thickening (Figure 3C). Podocyte foot process effacement was present in 10% of GBM surface. The proband has two sons, aged 36 (HR 2.2.) and 33 (HR 2.3.), who both inherited the same heterozygous mutation (Figure 2B). The older son (HR 2.2.) had hematuria with no proteinuria and preserved kidney function, while the younger (HR 2.3.) had no signs of the disease (no hematuria, no proteinuria, and preserved kidney function).

**Figure 4:**
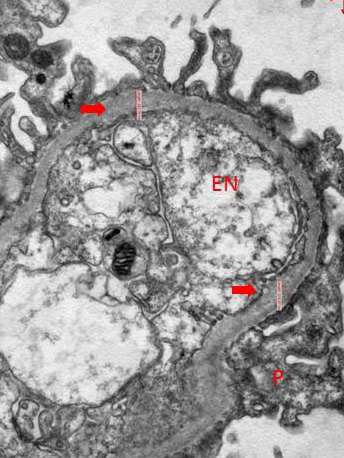
A kidney biopsy specimen of the proband HR 3.1. Glomerular capillary loop with glomerular basement membrane showing lamellation (arrows). EN – endothelium, P – podocyte. Transmission electron microscopy, magnification ×8000.

The third proband (HR 3.1.) was a 26-year-old man who presented with hematuria and proteinuria of 0.30 g in 24-hour urine, preserved kidney function (estimated glomerular filtration rate of 108 mL/min/1.73m^2^ by CKD-EPI equation), and hypertension. Tonal audiogram and eye exam did not reveal any changes characteristic for AS. LM showed five globally sclerosed glomeruli out of 26, and three with ischemic changes. There was no segmental sclerosis. Interstitial fibrosis and tubular atrophy were present in 10% of the cortical parenchyma. Blood vessels had normal morphology. Marked variations in GBM thickness (70-972 nm), with average thickness of 216 nm, were detected on EM. In the areas of thickening, lamellation and granular appearance of GBM were present (Figure 4). There was no podocyte foot process effacement.

**Figure 5:**
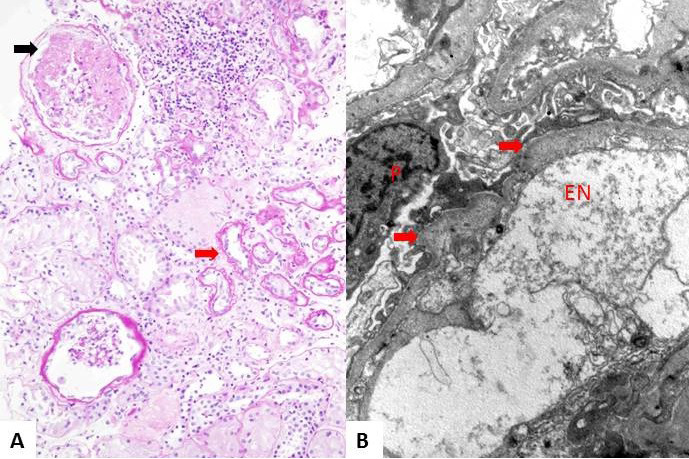
A kidney biopsy specimen of the proband HR 4.1. (**A**) The kidney cortex with a glomerulus, with segmental sclerosis (black arrow) and an area of interstitial fibrosis and tubular atrophy (red arrow). Periodic Acid-Schiff stain, magnification ×200. (**B**) A part of the glomerular capillary loop with focal lamellation of the glomerular basement membrane (arrow). EN – endothelium, P – podocyte. Transmission electron microscopy, magnification ×8000.

The fourth proband (HR 4.1.) was a 60-year-old woman who presented with hematuria and proteinuria of 2.30 g in 24-hour urine, preserved kidney function (estimated glomerular filtration rate of 110 mL/min/1.73 m^2^ by CKD-EPI equation), and hypertension. Perihilar FSGS was present in one out of six glomeruli on LM (Figure 5 A). One glomerulus was globally sclerosed. Interstitial fibrosis and tubular atrophy were present in 15% of the cortical parenchyma (Figure 5A). Arterioles showed mild hyalinosis, while arteries were not found in the kidney biopsy specimen. Thin GBM (144-288 nm, average thickness 205 nm) with focal lamellation was observed on EM (Figure 5B). Focal podocyte foot process effacement was present in 25% of the GBM surface. The proband's sister (age 53) was negative for the mutation.

**Figure 6:**
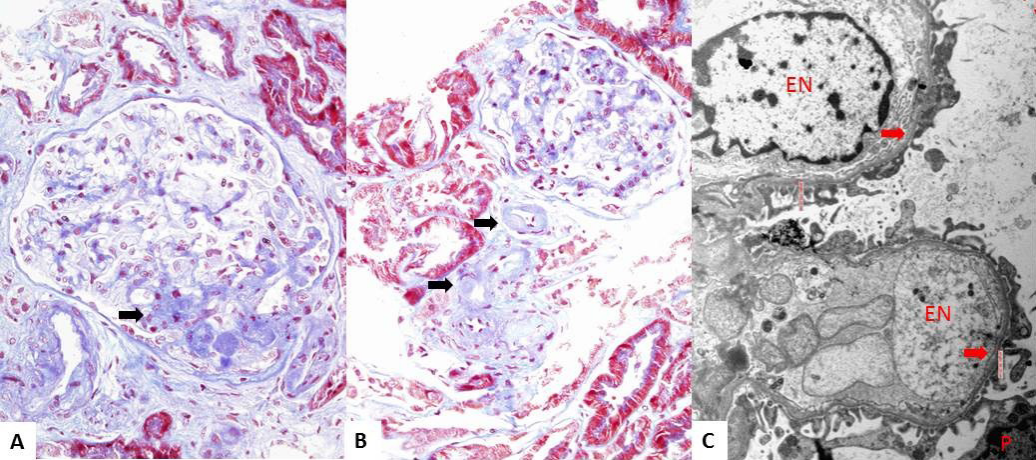
A kidney biopsy specimen of the proband HR 5.1. (**A**) A glomerulus with perihilar segmental sclerosis (arrow), Masson trichrome stain, magnification ×400. (**B**) Arteriolar hyalinosis (arrow). Masson trichrome stain, magnification ×200. (**C**) Glomerular capillary loops with thin glomerular basement membrane (arrow). EN – endothelium, P – podocyte. Transmission electron microscopy, magnification ×8000.

The fifth proband (HR 5.1.) was a 58-year-old woman who presented with hematuria, proteinuria of 1.14 g in 24-hour urine, moderately reduced kidney function (estimated glomerular filtration rate of 45 mL/min/1.73m^2^ by CKD-EPI equation), and hypertension. LM revealed perihilar FSGS (Figure 6A) in 23% of glomeruli and global glomerulosclerosis in 15% of glomeruli. Other glomeruli were enlarged. Interstitial fibrosis and tubular atrophy were present in 30% of the cortical parenchyma. Arterioles showed marked wall hyalinosis (Figure 6B), while arteries had normal morphology. Thin GBM (96-363 nm, average thickness 175 nm) was observed on EM (Figure 6C). There was no podocyte foot process effacement. The proband has a son (age 19) (HR5.2.) and daughter (age 38). The son, who is asymptomatic (no hematuria, no proteinuria, and preserved kidney function), inherited the same heterozygous mutation, while the daughter is negative for the mutation (Figure 2C).

### Genetic analysis

Next-generation sequencing (NGS) revealed splice site c.193-2A>C mutation in *COL4A4* at the genomic position chr2:227985866 in all patients. The youngest patient was homozygous, while other patients and family members were heterozygous for this mutation. This mutation has not been described previously in the Human Gene Mutation Database (HGMD), Leiden Open (source) Variation Database (LOVD), and Ensembl genome database (35). Bioinformatical analysis with Illumina VariantStudio software showed PVS1, PM2, and PP3 levels of certainty for pathogenicity according to the American College of Medical Genetics and Genomics (ACMG) (36). The described mutation was a null variant (within ±2 of canonical splice site) affecting gene *COL4A4* (PVS1), the allele was not found in GnomAD despite good coverage (PM2), and four pathogenic predictions, from DANN, GERP, MutationTaster, and scSNV-splicing, were present (PP3). The program scores were as follows: DANN score: 0.9909; GERP scores: NR 5.2399 and RS 5.2399; MutationTaster: accuracy 1 and coverted rankscore 0.8103; dbscSNV: ADA score 0.9999 and RF score 0.916. A comparison with an in-house database of 50 healthy individuals with NGS-sequenced *COL4A3*, *COL4A4,* and *COL4A5* genes also confirmed that it was a pathologic splice site variant c.193-2A>C in *COL4A4* at the genomic position chr2:227985866.

## Discussion

The main goal of our study was to present the pathohistological and clinical characteristics of five Croatian families with Alport spectrum disorders caused by splice acceptor pathogenic variant c.193-2A>C found in *COL4A4* at the genomic position chr2:227985866. One patient (the youngest, previously reported) was homozygous, while other patients were heterozygous for the mutation and presented with reasonably mild clinical phenotype and variable pathohistological findings (35). Not many potential splicing mutations have been identified within the first 10 nucleotides of the intron-exon boundaries for the *COL4A3* and *COL4A4* genes (37). Since this mutation has not been previously described in databases (HGMD, LOVD, and Ensembl genome database); since bioinformatical analysis showed PVS1, PM2, and PP3 levels of certainty for pathogenicity according to the ACMG; since the variant is not present among 50 healthy Croatian individuals; and since the characteristic pathohistological findings were found among the biopsied probands, we concluded this was a pathogenic variant causing autosomal Alport spectrum disorder (36). In the literature and databases, there are reports on *COL4A4* splice site mutation causing autosomal AS and TBMN (38,39). Rosado et al found IVS3 + 1G>C, replacement of guanine to cytosine in the position 1+ of intron 3, in the splicing region, suggesting ADAS with a mild phenotype in which kidney disease manifests at a later age without progression to ESRD (38). Xu et al reported on seven members of one family with TBMN and a heterozygous splicing mutation in *COL4A4* (c.1459 + 1G>A), resulting in the elimination of the entire exon 21 from the *COL4A4* cDNA and direct splicing of exons 20 and 22, which in turn caused a frameshift mutation after exon 20 in the open reading frame of *COL4A4* (39).

In a study by Savige et al, ARAS patients with *COL4A4* mutation had the mean age at ESRD onset of 25.4 ± 10.3 years (37). Our two-year-old homozygous patient had proteinuria and hematuria with normal kidney function and no extrarenal manifestations. For this patient, it is hard to predict the clinical course. He had EM changes characteristic of AS and only incipient chronic changes on LM.

Our heterozygous patients had a relatively benign clinical course. All of the biopsied probands presented with hematuria and proteinuria (ranging from 0.30 to 2.34 g in 24-hour urine). In addition, all of the biopsied probands were hypertensive. Only one heterozygous patient, aged 58, had a moderately reduced kidney function (estimated glomerular filtration rate 45 mL/min/1.73m^2^), while others had a preserved kidney function. Collagen IV nephropathy in heterozygous patients (TBMN) is characterized by incomplete penetrance, with normal findings in 5%-10% of heterozygous patients (40). Considering the type of mutation (c.193-2A>C in *COL4A4* at the genomic position chr2:227985866 with an expected result of whole exon skipping), we would have expected all heterozygous patients to have hematuria. However, the mother of the proband HR 1.1. (HR 1.3., age 35), the younger son of the proband HR 2.1. (HR 2.3., age 33), and the son of the proband 5.1. (HR 5.2., age 19) did not have hematuria. It is interesting to note that the father of the proband HR 1.1. (HR 1.2., age 37) was initially negative for hematuria, but was later found to be positive. According to the literature, some patients with TBMN have intermittent hematuria, which emphasizes the necessity of a continuous nephrological follow-up of heterozygous patients (41).

The pathohistological findings of our homozygous proband (HR 1.1.) revealed an increased number of immature or partially immature glomeruli for his age, with only incipient chronic changes on LM and EM changes characteristic for AS. Patients with AS have been shown to have an increased percentage of immature glomeruli (42,43). Heterozygous probands in our study, on the other hand, had variable kidney biopsy findings. Three patients (age 58-60) had TBMN on EM and FSGS on LM, two of them with focal lamellation on EM. Two patients had marked and one patient had mild arteriolar hyalinosis with interstitial fibrosis and tubular atrophy ranging from 10% to 30%. Interestingly, the youngest heterozygous patient (age 26) had changes characteristic for AS visible on EM with no FSGS observed on LM. He had no arteriolar hyalinosis, and only mild interstitial fibrosis and tubular atrophy were present (10%). Patients with collagen IV nephropathies, even within the same families, have highly variable phenotypic presentations. This is especially true for heterozygous patients (31). Heterozygous *COL4A3* or *COL4A4* patients with pathohistological changes characteristic for AS (lamellated GBM and GBM with basket-weave appearance on EM) have been described in the literature, and often referred to as ADAS (38,44-46). A limitation of our study is that we were not able to test our patients for further complicating genetic modifiers. The presence of the most severe EM changes of the GBM in our youngest heterozygous patient (aged 26 and with preserved kidney function and no extrarenal manifestations) suggests a potential influence of some modifying factors. Another limitation is that we could not exclude the possibility of deep intronic mutations, which are not detectable by NGS analysis, and compound heterozygosity (47). In the study by Dagher et al, 12% of individuals with TBMN in whom hematuria segregated with the *COL4A3* or *COL4A4* locus had hypertension (48). In our research, all biopsied probands were hypertensive. A perihilar variant of FSGS with glomerulomegaly has been reported in patients with systemic hypertension (49,50). One could argue that hypertension acted as a modifying factor of disease phenotype in all our biopsied probands (12).

In conclusion, while our only homozygous patient had evident clinical and histological signs of AS at a very young age, heterozygous patients presented with reasonably mild clinical phenotype and variable pathohistological findings at a later age. Our study shows variability of changes in pathohistological findings adding to the pool of knowledge about Alport spectrum disorders.
